# Conventional and regionally distinctive risk factors for first-onset myocardial infarction: the Bangladesh Risk of Acute Vascular Events (BRAVE) case–control study

**DOI:** 10.1016/j.lansea.2024.100519

**Published:** 2024-12-19

**Authors:** Rajiv Chowdhury, Aliya Naheed, Md Mostafa Monower, Sara Shahzad, Rubhana Raqib, Ishrat Tasmin, Sarah Spackman, Stephen Kaptoge, Lisa Pennells, Adam S. Butterworth, John Danesh, Emanuele Di Angelantonio

**Affiliations:** a Department of Global Health, Stempel College of Public Health and Social Work, Florida International University, Miami, FL, USA; b Initiative for Non Communicable Disease, Health Systems Studies Division, icddr,b, Mohakhali, Dhaka, Bangladesh; c Non Communicable Diseases, Nutrition Research Division, icddr,b, Mohakhali, Dhaka, Bangladesh; d Department of Epidemiology and Research, National Heart Foundation Hospital and Research Institute, Dhaka, Bangladesh; e British Heart Foundation Cardiovascular Epidemiology Unit, Department of Public Health and Primary Care, University of Cambridge, Cambridge, UK; f Infectious Diseases Division, icddr,b, Mohakhali, Dhaka, Bangladesh; g Victor Phillip Dahdaleh Heart and Lung Research Institute, University of Cambridge, Cambridge, UK; h British Heart Foundation Centre of Research Excellence, University of Cambridge, Cambridge, UK; i National Institute for Health and Care Research Blood and Transplant Research Unit in Donor Health and Behaviour, University of Cambridge, Cambridge, UK; j Health Data Research UK Cambridge, Wellcome Genome Campus and University of Cambridge, Cambridge, UK; k Department of Human Genetics, Wellcome Sanger Institute, Hinxton, UK; l Health Data Science Research Centre, Human Technopole, Milan, Italy

**Keywords:** Myocardial infarction, Risk factors, South Asia

## Abstract

**Background:**

South Asians may be particularly susceptible to premature myocardial infarction (MI) owing both to conventional cardiovascular risk factors and practices distinctive to South Asia. Identifying modifiable risk factors for MI in these populations could inform prevention strategies. We have, therefore, studied conventional risk factors and other characteristics in relation to occurrence of first MI in Bangladesh.

**Methods:**

In a case–control study involving 8133 first-onset MI cases and 8124 controls recruited in Bangladesh, we calculated odds ratios (ORs) for MI adjusted, for age, sex, smoking status, history of diabetes, history of hypertension, family history of MI, and LDL-cholesterol. We assessed the potential public health impact of risk factor modification using population attributable fractions (PAFs).

**Findings:**

The median (IQR) age of first MI was 53 (45–60) years. Adjusted ORs (95% CIs) were 2.80 (2.57–3.05) for cigarette smoking, 2.17 (1.94–2.43) for family history of MI, 2.27 (2.07–2.48) for history of hypertension, 1.91 (1.72–2.13) for history of diabetes, and 1.53 (1.47–1.58) per 1-SD higher LDL-cholesterol. The highest PAFs (95% CIs) were with current cigarette smoking (49% [46%–52%]), higher LDL-cholesterol (31% [29%–33%]) and history of hypertension (15% [13%–16%]). As for regionally distinctive practices, ORs were 4.02 (3.13–5.17) with *biri/hukkah* smoking, 2.09 (1.52–2.87) with chewing tobacco, and 1.26 (1.05–1.51) with parental history of first-cousin marriage.

**Interpretation:**

Our results confirm the relevance of several conventional risk factors to risk of first MI in Bangladesh, and identify associations with MI of practices distinctive to South Asia, including indigenous modes of tobacco consumption and parental first-cousin marriage. These findings suggest opportunities for cardiovascular disease prevention in Bangladesh that embrace both conventional and regionally distinctive risk factors.

**Funding:**

The BRAVE Study Coordinating Centre is underpinned by grants from the 10.13039/501100000274British Heart Foundation, UK Medical Research Council and National Institute for Health Research Cambridge Biomedical Research Centre.

**Research in context**

**Evidence before this study**

We searched MEDLINE for articles published in English from inception to September 30, 2024, reporting associations between conventional or regionally distinctive risk factors and myocardial infarction (MI) in South Asian populations. Search terms related to exposures (tobacco use, smoking, hypertension, diabetes, lipids), regionally specific practices (bidi smoking, chewing tobacco, first-cousin marriage), study design (case–control, cohort), and association measures (odds ratios, risk ratios, population-attributable fractions). Although several studies have assessed conventional cardiovascular risk factors like smoking, diabetes, and cholesterol in South Asians, only a handful have explored the combined relevance of these factors with indigenous practices common in South Asia, such as smokeless tobacco use and consanguinity, in large-scale, well-powered studies.

**Added value of this study**

The BRAVE study provides the largest dataset to date on the risk factors for first-onset MI in Bangladesh. With over 8000 cases and 8000 controls, this study examines both conventional risk factors—such as smoking, hypertension, and elevated LDL cholesterol—and regionally distinctive practices, including the use of bidi/hukkah and chewing tobacco, as well as first-cousin marriage. The study confirms the relevance of conventional risk factors but also suggested that some practices distinctive to South Asia are associated with MI risk. For instance, bidi/hukkah smoking is associated with a fourfold increase in MI risk, and chewing tobacco is linked to a twofold increase in risk. These findings expand the understanding of cardiovascular risk in South Asia and highlight the need for context-specific public health strategies.

**Implications of all the available evidence**

This study underscores the importance of addressing both conventional and regionally distinctive cardiovascular risk factors to reduce the burden of MI in Bangladesh and other South Asian countries. The significant contributions of smoking (particularly bidi/hukkah) and chewing tobacco to MI risk suggest that tobacco control strategies should extend beyond cigarette smoking to include indigenous forms of tobacco use. The association between first-cousin marriage and MI also points to a need for further investigation into genetic and cultural factors in cardiovascular disease risk. Overall, these findings support the development of targeted prevention strategies that incorporate the region's unique risk factor profile and promote cardiovascular health in South Asia.

## Introduction

For public health and scientific reasons, there is a need to understand why South Asians are at unusually high risk of premature coronary heart disease (CHD),^1^^,^^2^ which accounts for a substantial number of life-years lost, mainly as a consequence of South Asia's large population (∼2 billion people^3^) and the region's high CHD death rates at younger ages.^4^, ^5^, ^6^ In contrast with age-standardised reductions observed in CHD in many high- and middle-income countries over recent decades, South Asia's high CHD mortality rates have been increasing.^7^ For example, Bangladesh is a lower-middle-income country of 170 million people that has in recent decades made considerable progress toward UN Millennium Development Goals.^8^ However, as the country has also experienced large increases in the burden of non-communicable diseases (especially cardiovascular diseases), it threatens to undermine health and economic gains.^9^^,^^10^ CHD now accounts for a substantial proportion of all adult deaths annually in Bangladesh as well as being a major contributor to disability, lost earnings and social dislocation. Excess CHD burdens also apply to the several million immigrants of Bangladeshi descent living in industrialised countries.^11^, ^12^, ^13^

Although several conventional vascular risk factors (such as cigarette smoking, diabetes mellitus, and higher levels of LDL-cholesterol and blood pressure) are common among South Asians,^14^ their relative and absolute attributions to first MI risk are not precisely known. Additionally, relatively few well-powered studies have had the ability to investigate concomitantly the relevance of both conventional cardiovascular and regionally distinctive risk factors that are widely prevalent across South Asia, including indigenous forms of tobacco use (e.g., *biri*, *jarda* or *gul*) and distinctive practices (e.g., first-cousin marriage).^14^

We, therefore, report findings from a case–control study in Bangladesh involving 8133 patients with first occurrence of myocardial infarction and 8124 controls, focusing on associations of conventional risk factors and several characteristics and practices distinctive to South Asia.

## Methods

### Participants

Details of the Bangladesh Risk of Acute Vascular Events (BRAVE) study have been described in detail elsewhere.^15^ Briefly, between 2011 and 2016 BRAVE enrolled a total of 8133 cases of acute first MI and 8124 controls frequency-matched to cases by sex and age in 5-year age groups. MI cases were patients aged >20 years admitted to emergency rooms of the collaborating national cardiology referral hospital in Dhaka. Patients were eligible for inclusion as MI cases if they had all of the following characteristics: (1) sustained symptoms suggestive of MI lasting longer than 20 min within the previous 48 h, (2) ECG changes indicative of MI, (3) positive troponin test, (4) no previous cardiovascular diseases (CVD), defined as self-reported history of angina, MI, coronary revascularisation, transient ischaemic attack, stroke, or evidence of CHD on accessible prior ECG or other medical records, (5) no previous history of other chronic diseases, defined as self-reported chronic kidney disease, liver disease, chronic obstructive pulmonary disease, or cancer, and (6) not concurrently hospitalised for any other CVD events. Controls were individuals without a previous self-reported history of CVD concurrently identified in the same hospital as index cases, and recruited in the following order of priority: visitors of patients attending the outpatient department, visitors of in-patients who were not part of the BRAVE study, and visitors of index MI cases who were not their blood relatives.

### Questionnaire administration and physical measurements

Trained medical research officers administered locally-developed and pre-piloted questionnaires that: sought information on demographic characteristics (e.g., current residence, income, occupation, and marital history), personal and family (parental) medical history, behavioural (e.g., tobacco use, dietary intake, physical activity) and societal factors (e.g., intermarriage). For MI cases, data were obtained from the participants after medical stabilisation or a close family member, and enquired about behaviours and characteristics during or before the time of diagnosis of acute MI.

Diabetes was defined by self-report, medication usage, and/or HbA1c ≥ 6.5%. Only history of hypertension (defined by self-report and/or medication usage) was used in the analysis because blood pressure levels may be lowered by acute MI or its treatment. To collect information on smoking patterns distinctive to South Asian populations, participants answered a population-specific questionnaire used in other studies among South Asians.^16^ This included questions on consumption of chewable tobacco (e.g., *gul, jarda, tamak* and *khoyer*) and non-tobacco products (e.g., *betel leaves/paan, betel nuts/supari*), conventional inhalation tobacco (e.g., cigarettes), and local inhalation tobacco products (e.g., *biri* and *hukka*).

To assess dietary habits, the BRAVE study adapted a purpose-designed food-frequency questionnaire (FFQ), as described elsewhere,^15^^,^^17^ which estimated standard portion size assigned to each food item. Food quantities per day (g/d) were calculated by multiplying the portion size of the FFQ items by the relevant multiplier, and then items were grouped based on nutrient content and local culinary usage. Total energy intake per day (kcal/d) was calculated by multiplying the frequency of consumption of each FFQ item by the energy content of the standard portion size, divided by 100, based on the nutrition composition table from Bangladesh.^18^ Physical activity was based on a validated International Physical Activity Questionnaire^19^ that evaluated activity at work, travel, recreational activities and sedentary behaviour. Frequency and intensity of these activities on a typical day were calculated in terms of metabolic equivalents or METs/min/week by multiplying the average energy expenditure (3.3 MET for walking, 4.0 MET for moderate intensity, and 8.0 MET for vigorous intensity) by min/week for each physical activity. Information was sought from participants about whether they, or their parents, were married to a first-cousin. Level of stress was defined as high/severe if the participant reported experiencing stress almost always during the two weeks before the MI event. Daytime nap was assessed by asking the number of hours the participant slept during daytime, on an average, over the past three months. The poverty line was defined according to World Health Organization criteria (ie, individuals living on less than $1.90 per day, or ∼56,000 Taka per day).

### Collection of biological samples and laboratory procedures

Non-fasting blood samples were obtained and centrifuged within 45 min of collection. In MI cases, blood sampling was within 48 h of the onset of symptoms and prior to administration of any thrombolytic agents. Laboratory personnel unaware of case–control status measured total-, HDL- and LDL-cholesterol using enzymatic assays. Assay plates contained a mixture of cases and controls, including negative and positive controls.

### Statistical analyses

Odds ratios (ORs) were calculated using unconditional logistic regression, adjusted, unless otherwise specified, for age, sex, tobacco use, LDL-cholesterol, history of diabetes, history of hypertension, waist-hip ratio, and family history of MI at any age. To assess shapes of associations, ORs were calculated according to fifths (for food groups) or tenths of risk factors and plotted against the group-specific mean risk factor values. 95% CIs were calculated from the variances that reflect the amount of information underlying each group (including the reference group).^20^ When a linear dose–response relationship was appropriate, we calculated the adjusted ORs per 1 standard deviation (SD) higher for continuous variables. We investigated possible effect-modification by age, sex and place of residence using tests of interactions and emphasised findings with p < 0.001.

We assessed public health impact by calculating population attributable fractions (PAF) using methods appropriate for a case–control study, analysed using multivariable logistic regression.^21^ For the simple case of a binary exposure variable, $PAF=p_{E|cases}\times \left(OR-1\right)/OR$, where $p_{E|cases}$ is the prevalence of exposure in cases and OR is the crude odds ratio for association of exposure and MI. The multivariable logistic regression method allowed calculation of PAF based on adjusted associations for different exposure variables and MI, comparing an idealised (e.g., exposure absent) vs observed scenario. Confidence intervals for the PAFs were calculated from fitted model parameters after applying normalising and variance stabilising transformations as described in the software methods paper.^21^ Main findings are based on complete case analysis; sensitivity analysis with imputation of missing data using the multivariate imputation by chained equations (MICE) method repeated 20 times are presented in the Supplementary Material. The missing data imputation was stratified by the observed outcome (i.e., MI case–control status) and included all the risk factors analysed. Continuous variables were imputed using linear regression, binary variables using logistic regression and categorical variables using multinomial logistic regression. All analyses were performed using Stata (version 17, StataCorp, College Station, TX, USA).

### Ethical approval and informed consent

The study has received approval from the relevant research ethics committee of each of the institutions involved in participant recruitment. Written informed consent was obtained from each participant prior to recruitment. Data collected in this research are subject to the core data protection principles and requirements of the UK Data Protection Act 1998. The investigators and institutional review boards are committed to ensure that research is conducted according to the latest version of the Declaration of Helsinki, the Universal Declaration on the Human Genome and Human Rights adopted by UNESCO, and other relevant legislation.

### Role of the funding source

The funders of the study had no role in study design, data collection, data analysis, data interpretation, or writing of the report.

## Results

### Participants characteristics

Median age (IQR) of MI cases was 53 (45–60) years; 2824 (36%) cases were younger than 50 years; 7127 (88%) were male; 2213 (32%) cases and 2376 (30%) controls were urban residents, whereas the rest were from either semiurban or rural areas. Compared to the control group, MI cases had higher prevalence of diabetes (1545 [19%] vs 837 [10%]), hypertension (2170 [27%] vs 1104 [14%]), and a family history of MI (1114 [14%] vs 583 [7%]). MI cases were also more likely to be current tobacco users (5436 [77%] vs 4895 [61%]), and have no formal education (2643 [38%] vs 2817 [35%]; Table 1).

**Table 1:** Participant characteristics by myocardial infarction (MI) case–control status.

Characteristics	N	MI cases	N	Controls
		Median (IQR) or n (%)		Median (IQR) or n (%)
**Socio-demographic factors**				
Age (yrs)	8133	53.0 (45.0, 60.0)	8124	51.0 (44.0, 58.0)
Male sex	8133	7127 (88%)	8124	7116 (88%)
Location	6995		8052	
Urban		2213 (32%)		2376 (30%)
Semi-urban		1092 (16%)		1249 (16%)
Rural		3690 (53%)		4427 (55%)
Education	7028		8101	
No formal education		2643 (38%)		2817 (35%)
Primary		2142 (30%)		2407 (30%)
Secondary		1453 (21%)		1923 (24%)
Vocational/University		790 (11%)		954 (12%)
Occupation	7056		8101	
Business/professional		3236 (46%)		3560 (44%)
Industrial worker		495 (7%)		438 (5%)
Manual labour		1014 (14%)		1675 (21%)
Unemployed/retired/student		2311 (33%)		2428 (30%)
Annual income below poverty line^a^	6985	1834 (26%)	8064	1921 (24%)
Parental intermarriage	6983	284 (4%)	8076	274 (3%)
**Clinical history**				
History of diabetes	8123	1545 (19%)	8120	837 (10%)
History of hypertension	8122	2170 (27%)	8120	1104 (14%)
Family history of MI	8133	1114 (14%)	8124	583 (7%)
Waist-to-hip ratio	6689	0.97 (0.93, 1.02)	8119	0.96 (0.91, 1.01)
**Behavioural factors**				
Tobacco consumption	7071		8084	
Never		1171 (17%)		2598 (32%)
Former		464 (7%)		591 (7%)
Current		5436 (77%)		4895 (61%)
Amount of physical activity	8133		8124	
<600 MET/wk		3452 (42%)		1872 (23%)
≥600 MET/wk		4681 (58%)		6252 (77%)
**Blood lipids**				
Total cholesterol (mmol/l)	8118	5.01 (4.33, 5.76)	8115	4.71 (4.13, 5.39)
LDL-C (mmol/l)	8118	3.16 (2.57, 3.84)	8115	2.77 (2.23, 3.35)
HDL-C (mmol/l)	8118	0.80 (0.67, 0.95)	8115	0.82 (0.69, 0.98)

IQR, interquartile range; MI, myocardial infarction; MET, metabolic equivalents; HDL-C, high-density lipoprotein cholesterol; LDL-C, low-density lipoprotein cholesterol.

a Less than 56,000 Taka according to World Health Organization defined poverty line ($1.90/day).

### Association of conventional risk factors with MI

**Fig. 1:**
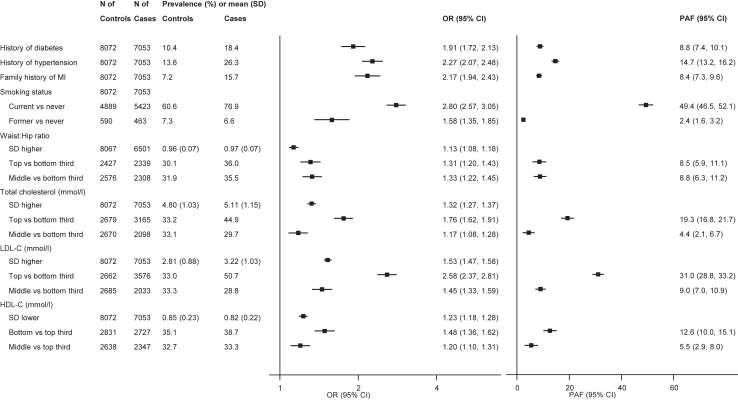
Prevalences, absolute and relative risk estimates for various conventional risk factors with myocardial infarction (MI). Analysis adjusted for age, sex, tobacco use, LDL-cholesterol, history of diabetes, history of hypertension, and family history of MI. SD, standard deviation; HDL-C, high-density lipoprotein cholesterol; LDL-C, low-density lipoprotein cholesterol.

**Fig. 2:**
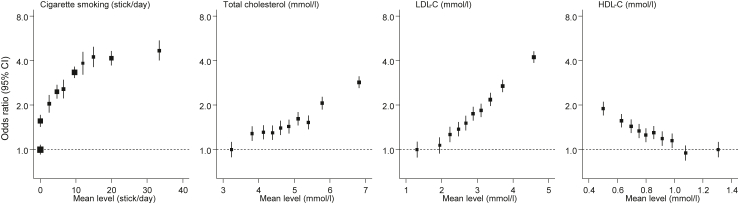
Shape of associations for various conventional risk factors with myocardial infarction (MI). Analysis adjusted for age, sex, tobacco use, LDL-cholesterol, history of diabetes, history of hypertension, and family history of MI; HDL-C, high-density lipoprotein cholesterol; LDL-C, low-density lipoprotein cholesterol.

**Fig. 3:**
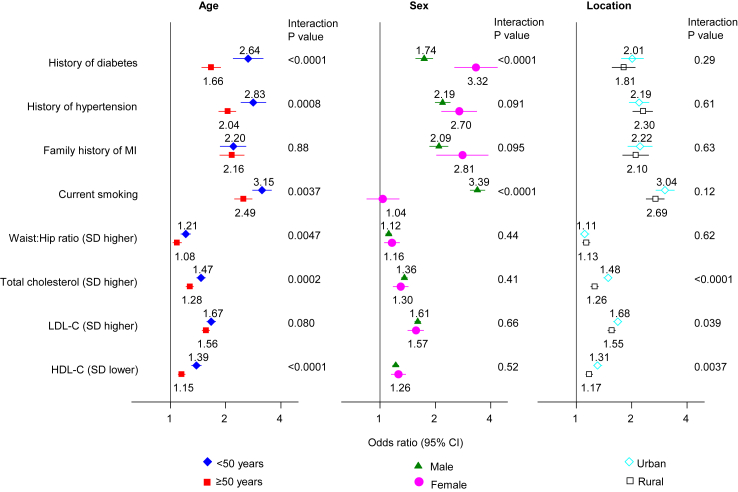
Associations of various conventional risk factors with myocardial infarction (MI), stratified by age, sex and location. Analysis adjusted for age, sex, tobacco use, LDL-cholesterol, history of diabetes, history of hypertension, and family history of MI; HDL-C, high-density lipoprotein cholesterol; LDL-C, low-density lipoprotein cholesterol.

Adjusted ORs and corresponding 95% CIs were 1.91 (1.72–2.13) with history of diabetes, 2.27 (2.07–2.48) with history of hypertension, and 2.17 (1.94–2.43) with family history of MI (Fig. 1). Compared with never cigarette smokers, OR for MI was 2.80 (2.57–3.05) among current cigarette smokers (Fig. 1), with an increasing dose–response relationship observed according to numbers of cigarettes smoked per day, at least up to about 20 cigarettes smoked per day (Fig. 2). There were also approximately log-linear associations of MI risk with total, LDL- and HDL-cholesterol (the latter being an inverse association; Fig. 2). Adjusted ORs per 1-SD higher waist-to-hip ratio, total cholesterol, and LDL-cholesterol were 1.13 (1.08–1.18), 1.32 (1.27–1.37), and 1.53 (1.47–1.58), respectively, and 1.23 (1.18–1.28) per 1-SD *lower* HDL-cholesterol (Fig. 1). Subgroup explorations suggested that lipid levels, history of diabetes and history of hypertension were potentially more strongly associated with risk of MI among younger individuals (p < 0.001; Fig. 3, eTable 1). MI association with history of diabetes was potentially stronger in women than men (Fig. 3, eTable 2); the association of total cholesterol with MI was potentially stronger in individuals resident in urban than rural areas (Fig. 3, eTable 3).

### Population attributable fractions for conventional risk factors

Fig. 1 presents estimated overall PAFs for various conventional risk factors. The potential relative importance of risk factors to public health varied, broadly relating to the prevalence of risk factors among MI cases. The PAFs (95% CI) were: 49% (47%–52%) with current cigarette smoking; 31% (29%–33%) with higher LDL-C (top third); and 15% (13%–16%) with history of hypertension. By contrast, the corresponding values were generally lower for former smoker, history of diabetes, and family history of MI (PAF <10% for each, Fig. 1).

### Association of regionally distinctive characteristics and practices with first-ever MI risk

**Fig. 4:**
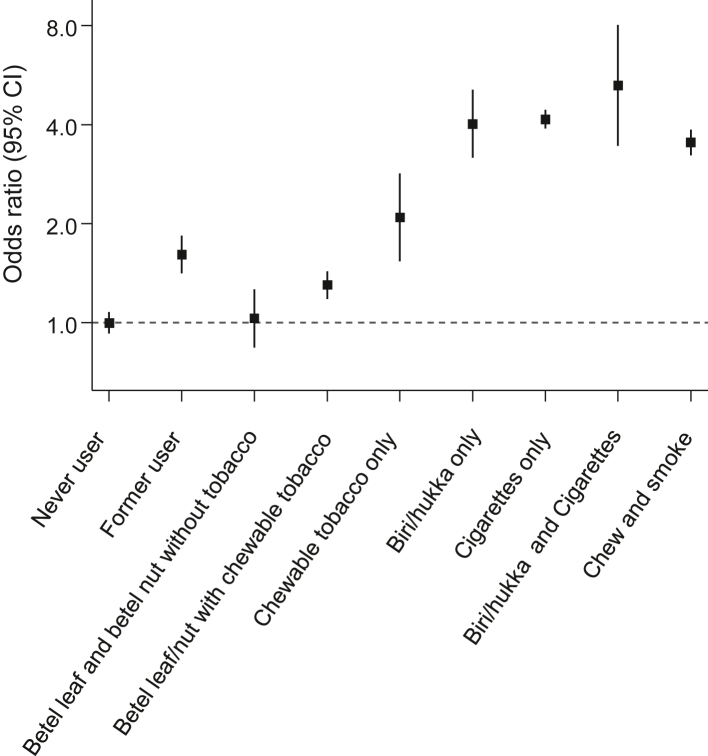
Local patterns of tobacco consumption in relation to myocardial infarction (MI). Analysis adjusted for age, sex, tobacco use, LDL-cholesterol, history of diabetes, history of hypertension, and family history of MI.

Compared with never users of any form of tobacco (smoking or chewing), ORs (95% CIs) were 4.02 (3.13–5.17) for inhalation smoking (*biri*/*hukka*), 2.09 (1.52–2.87) for chewing tobacco (*gul/jarda/tamak/khoyer*) and 4.15 (3.76–4.59) for cigarette smoking only (Fig. 4). The corresponding OR was 1.03 (0.83–1.28) in individuals who chewed *paan* (betel leaf) and *supari* (betel nut) without concomitant tobacco products (e.g., *jarda*), and 1.30 (1.15–1.47) when chewed *paan* and *supari* included tobacco. Finally, the OR was 5.26 (3.42–8.09) in people who smoked both tailor-made cigarettes and indigenous *biri/hukka*, and it was 3.54 (3.13–3.99) in those who smoked as well as chewed tobacco (Fig. 4).

As regards other characteristics, ORs for MI were: 1.26 (1.05–1.51) among individuals with a parental history of intermarriage; 1.38 (1.22–1.56) for people who were lifelong residents in urban vs rural areas; 1.29 (1.19–1.39) for people with <5 years of educational attainment; 1.25 (1.13–1.38) for individuals whose income was below the poverty line; 1.37 (1.25–1.50) in people who reported high stress at work; and 1.17 (1.08–1.26) in people who reported taking any day-time naps (eFig. 1).

Higher levels of weekly physical activity (i.e., work, travel or sports-based) were also associated with lower risk of MI. Additionally, in analyses investigating dose–response associations according to hours spent sitting per day, a suggestive J-shaped relationship was observed with a broadly linear dose–response beyond 2 h of sitting (eFig. 2). For major food groups with the highest average daily consumption, ORs for MI (adjusted for conventional CVD factors, level of education and total energy intake), comparing the top vs the bottom fifths of dietary intake, were: 1.02 (0.90–1.15) for rice; 1.00 (0.98–1.12) for red meat; 0.90 (0.81–1.01) for white meat; 1.55 (1.37–1.77) for fish; 1.25 (1.12–1.40) for total oil (principally soybean and sunflower); 0.52 (0.46–0.59) for fruit; and 0.62 (0.55–0.70) for vegetable consumption (eFig. 3).

Findings were broadly similar to those described above in sensitivity analyses that involved all 8133 MI cases and 8124 controls after multiple imputation of missing data (eFigs. 4–7).

## Discussion

This study has helped characterise major drivers of MI in Bangladesh, yielding findings with potential implications for disease understanding and control. First, our results have shown that several well-established causal cardiovascular risk factors (eg, smoking, blood pressure, lipids, diabetes, obesity) contribute importantly to risk of first MI in Bangladesh. For example, our findings have demonstrated ─ probably for the first time in a Bangladeshi population ─ strong and nearly log-linear relationships of MI risk with number of cigarettes smoked and with concentration of pro-atherogenic lipids.

Second, our results suggest that the associations of MI with some conventional risk factors can vary substantially by context.^22^ For example, our study has suggested that the proportional impact of lipids and diabetes mellitus on MI might be greater in younger adults, meaning younger Bangladeshis may be particularly vulnerable to the cardiovascular consequences of metabolic dysfunction. Our findings have also suggested that diabetes may be a stronger risk factor in women than men in Bangladesh.

Third, compared with population attributable fractions (PAF) for risk factors reported for other global regions,^23^ PAFs estimated in our Bangladesh study were higher with smoking and pro-atherogenic lipids, whereas PAFs were lower with diabetes, hypertension and waist-to-hip ratio. It is unclear to what extent these apparent cross-population differences in PAFs could reflect differences in study design, exposure heterogeneity (eg, tobacco consumption patterns, such as higher biri and smokeless tobacco use in South Asia), healthcare variations (e.g., lower access to statins in South Asia), some combination of these factors, or other characteristics. However, if further studies can confirm differences in PAFs, it would strengthen the rationale for context-specific public health interventions.^24^

Fourth, our results suggest that, in addition to conventional risk factors, certain practices distinctive to South Asia may contribute to MI risk in Bangladesh. For example, our results have indicated that use of smokeless tobacco products, such as tobacco chewing,^25^^,^^26^ is associated with MI risk,^27^ reinforcing previous suggestions^28^ that cardio-toxic elements in tobacco, such as nitrosamines, can lead to endothelial dysfunction and promote atherosclerosis.^29^^,^^30^ Furthermore, our findings have suggested that there was a 4-fold relative risk of MI with inhalation smoking, which was at least as strong as the relative risk we observed with cigarette smoking, highlighting mechanisms proposed to mediate the effects of *biri*/*hukka* (e.g., oxidative stress, thrombogenesis, and systemic inflammation^31^, ^32^, ^33^) and underscoring the importance of extending tobacco control strategies to smokeless, chewable, and forms of tobacco products.^29^^,^^30^ We also found that parental first-cousin marriage was associated with MI risk, suggesting the need for future studies to determine whether this association principally reflects residual confounding (e.g., by poorly measured factors related to socioeconomic disadvantage) or causality. In case of the latter, intermarriage could constitute a potentially modifiable practice, for which it has been suggested that genetic counselling could play a role in communities with high rates of consanguinity to reduce the risk of recessive diseases.^34^^,^^35^

Finally, our exploratory analysis of multiple characteristics implicated in CHD in Western populations may encourage further study of some of these factors in other South Asian studies.^36^, ^37^, ^38^, ^39^, ^40^, ^41^, ^42^ For example, in contrast with reports in most Western populations, we found surprising associations between higher fish consumption and *higher* MI risk, possibly explained by widespread prevalence of arsenic-contaminated waters, and local practices, including use of formalin to preserve fish and prolonged and deep frying of fish with re-used cooking oils.^43^, ^44^, ^45^

The strengths and potential limitations of this study merit consideration. Our study had substantial statistical power, involving five times as many CHD events as in the previous largest study of first MI in South Asia.^6^ We investigated a range of conventional and other risk factors, extending from biochemical to behavioural factors, using locally developed and pre-piloted instruments. We validated MI cases using standardised criteria. We implemented several measures to help limit potential biases in our case–control study.^16^^,^^23^^,^^46^ For example, to help reduce selection bias, we sampled controls from approximately the same source population as the cases. To reduce the scope for recall bias, we conducted an incident case–control study of acute MI and sought information from cases within hours of the index event. Recall bias is a potential limitation in this study, particularly for self-reported behaviours such as smoking, diet, and physical activity. However, we aimed to mitigate this bias by collecting data as soon as possible after MI diagnosis, minimising the likelihood of differential recall between cases and controls. Additionally, we used standardised questionnaires, which have been validated in similar populations, to reduce the variability in self-reported information. Nevertheless, missing values on some variables was inevitable, with some MI patients unable to provide all information either due to early death or measurements being infeasible. However, sensitivity analyses using multiple imputation supported the findings from the complete case analysis. Further studies will seek to evaluate and extend the current findings by using complementary methods, including large-scale long-term cohort studies, which we and others are establishing in South Asia.^47^

In conclusion, our study has confirmed the relevance of several conventional risk factors to risk of first MI in Bangladesh. We also identified associations with MI of practices distinctive to South Asia, including indigenous modes of tobacco consumption and parental first-cousin marriage, underscoring the potential importance of identifying context-specific strategies for cardiovascular disease prevention in South Asia that embrace both conventional and regionally distinctive risk factors.

## Contributors

RC, AN, MM, RR, JD and EDA conceived and designed the study. MM, IT, SSp collected and verified the underlying data. RC, SSh, SK, and LP did the analyses. RC, AN, SSh, JD and EDA drafted the manuscript. All authors acquired and interpreted the data, critically revised the paper, and had final responsibility for the decision to submit for publication.

## Data sharing statement

Data are available upon application to the study's Steering and Data Access Committee.

Codes are available upon request.

## Declaration of interests

John Danesh holds a British Heart Foundation Professorship and an NIHR Emeritus Senior Investigator Award [∗]. John Danesh serves on scientific advisory boards for AstraZeneca, Novartis, and UK Biobank, and has received multiple grants from academic, charitable and industry sources outside of the submitted work. Emanuele Di Angelantonio holds an NIHR Senior Investigator Award [∗]. Adam Butterworth reports institutional grants from AstraZeneca, Bayer, Biogen, BioMarin, Bioverativ, Novartis, Regeneron and Sanofi.

∗The views expressed are those of the authors and not necessarily those of the NIHR or the Department of Health and Social Care.

## Appendix A Supplementary data

eFigures and eTables
