# Supplementary material for: Conventional and regionally distinctive risk factors for first-onset myocardial infarction: the Bangladesh Risk of Acute Vascular Events (BRAVE) case–control study
Source: Lancet Reg Health Southeast Asia. 2024 Dec 19;32:100519. doi: 10.1016/j.lansea.2024.100519 (PMC11730263; doi:10.1016/j.lansea.2024.100519)
Supplement: eFigures and eTables [file mmc1.pdf]

Supplementary material

Conventional and regionally distinctive risk factors for first-onset myocardial infarction: The Bangladesh Risk of Acute Vascular Events (BRAVE) study

eTable 1. Participants characteristics by age group and MI case-control status 2

eTable 2. Participants characteristics by sex and MI case-control status 3

eTable 3. Participants characteristics by location and MI case-control status 4

eFigure 1. Associations of additional factors with MI 5

eFigure 2. Physical activity and risk of MI 6

eFigure 3. Association of food groups with MI 7

eFigure 4. Sensitivity analysis with multiple imputation of missing data: Prevalences, absolute and relative risk estimates for various conventional risk factors with MI......... 8

eFigure 5. Sensitivity analysis with multiple imputation of missing data: Associations of various conventional risk factors with MI, stratified by age, sex and location............... 9

eFigure 6. Sensitivity analysis with multiple imputation of missing data: Associations of additional factors with MI..10

eFigure 7. Sensitivity analysis with multiple imputation of missing data: Associations of food groups with MI. 11

eTable 1. Participants characteristics by age group and MI case-control status

	<50 years				≥50 years			
	MI Cases		Controls		MI Cases		Controls	
Characteristics	N	Median (IQR) or n (%)	N	Median (IQR) or n (%)	N	Median (IQR) or n (%)	N	Median (IQR) or n (%)
**Socio-demographic factors**								
Age (yrs)	2824	43.0 (38.0, 45.0)	3425	43.0 (38.0, 46.0)	5309	58.0 (53.0, 65.0)	4699	57.0 (53.0, 62.0)
Male sex	2824	2569 (91%)	3425	3112 (91%)	5309	4558 (86%)	4699	4004 (85%)
Location	2533		3396		4462		4656	
Urban	2533	962 (38%)	3396	1141 (34%)	4462	1251 (28%)	4656	1235 (27%)
Semi-urban	2533	470 (19%)	3396	594 (17%)	4462	622 (14%)	4656	655 (14%)
Rural	2533	1101 (43%)	3396	1661 (49%)	4462	2589 (58%)	4656	2766 (59%)
Education	2539		3417		4489		4684	
No formal education	2539	678 (27%)	3417	847 (25%)	4489	1965 (44%)	4684	1970 (42%)
Primary	2539	824 (32%)	3417	1035 (30%)	4489	1318 (29%)	4684	1372 (29%)
Secondary	2539	617 (24%)	3417	948 (28%)	4489	836 (19%)	4684	975 (21%)
Vocational/University	2539	420 (17%)	3417	587 (17%)	4489	370 (8%)	4684	367 (8%)
Occupation	2546		3418		4510		4683	
Business/professional	2546	1450 (57%)	3418	1898 (56%)	4510	1786 (40%)	4683	1662 (35%)
Industrial worker	2546	283 (11%)	3418	247 (7%)	4510	212 (5%)	4683	191 (4%)
Manual labour	2546	283 (11%)	3418	599 (18%)	4510	731 (16%)	4683	1076 (23%)
Unemployed/retired/student	2546	530 (21%)	3418	674 (20%)	4510	1781 (39%)	4683	1754 (37%)
Annual income below poverty line‡	2528	341 (13%)	3409	465 (14%)	4457	1493 (33%)	4655	1456 (31%)
Parental intermarriage	2527	101 (4%)	3407	126 (4%)	4456	183 (4%)	4669	148 (3%)
**Clinical history**								
History of diabetes	2820	446 (16%)	3423	213 (6%)	5303	1099 (21%)	4697	624 (13%)
History of hypertension	2820	636 (23%)	3423	325 (9%)	5302	1534 (29%)	4697	779 (17%)
Waist-to-hip ratio	2427	0.97 (0.93, 1.01)	3423	0.96 (0.91, 0.99)	4262	0.98 (0.93, 1.02)	4696	0.96 (0.91, 1.02)
**Lifestyle factors**								
Tobacco consumption	2558		3412		4513		4672	
Never	2558	555 (22%)	3412	1495 (44%)	4513	616 (14%)	4672	1103 (24%)
Ex	2558	86 (3%)	3412	160 (5%)	4513	378 (8%)	4672	431 (9%)
Current	2558	1917 (75%)	3412	1757 (51%)	4513	3519 (78%)	4672	3138 (67%)
Amount of physical activity	2824		3425		5309		4699	
<600 MET/wk	2824	1157 (41%)	3425	877 (26%)	5309	2295 (43%)	4699	995 (21%)
>=600 MET/wk	2824	1667 (59%)	3425	2548 (74%)	5309	3014 (57%)	4699	3704 (79%)
**Blood lipids**								
Total cholesterol (mmol/l)	2819	5.16 (4.45, 5.94)	3418	4.72 (4.13, 5.40)	5299	4.93 (4.27, 5.66)	4697	4.71 (4.14, 5.38)
LDL-C (mmol/l)	2819	3.19 (2.60, 3.93)	3418	2.73 (2.20, 3.31)	5299	3.15 (2.55, 3.79)	4697	2.81 (2.27, 3.38)
HDL-C (mmol/l)	2819	0.78 (0.66, 0.92)	3418	0.82 (0.68, 0.97)	5299	0.81 (0.68, 0.97)	4697	0.83 (0.69, 0.99)

IQR, interquartile range; MI, myocardial infarction; MET, metabolic equivalents; HDL-C, high-density lipoprotein cholesterol; LDL-C, low-density lipoprotein cholesterol.

‡Less than 56,000 Taka according to World Health Organization defined poverty line ($1.90/day); 1 Taka = $0.012.

eTable 2. Participants characteristics by sex and MI case-control status

	Male				Female			
	MI Cases		Controls		MI Cases		Controls	
Characteristics	N	Median (IQR) or n (%)	N	Median (IQR) or n (%)	N	Median (IQR) or n (%)	N	Median (IQR) or n (%)
**Socio-demographic factors**								
Age (yrs)	7127	52.0 (45.0, 60.0)	7116	51.0 (44.0, 58.0)	1006	55.0 (49.0, 65.0)	1008	55.0 (47.0, 60.0)
Male sex	7127	7127 (100%)	7116	7116 (100%)	1006	0 (0%)	1008	0 (0%)
Location	6219		7051		776		1001	
Urban	6219	2021 (32%)	7051	2131 (30%)	776	192 (25%)	1001	245 (24%)
Semi-urban	6219	973 (16%)	7051	1117 (16%)	776	119 (15%)	1001	132 (13%)
Rural	6219	3225 (52%)	7051	3803 (54%)	776	465 (60%)	1001	624 (62%)
Education	6245		7096		783		1005	
No formal education	6245	2145 (34%)	7096	2234 (31%)	783	498 (64%)	1005	583 (58%)
Primary	6245	1926 (31%)	7096	2125 (30%)	783	216 (28%)	1005	282 (28%)
Secondary	6245	1400 (22%)	7096	1819 (26%)	783	53 (7%)	1005	104 (10%)
Vocational/University	6245	774 (12%)	7096	918 (13%)	783	16 (2%)	1005	36 (4%)
Occupation	6268		7097		788		1004	
Business/professional	6268	3207 (51%)	7097	3484 (49%)	788	29 (4%)	1004	76 (8%)
Industrial worker	6268	480 (8%)	7097	425 (6%)	788	15 (2%)	1004	13 (1%)
Manual labour	6268	999 (16%)	7097	1638 (23%)	788	15 (2%)	1004	37 (4%)
Unemployed/retired/student	6268	1582 (25%)	7097	1550 (22%)	788	729 (93%)	1004	878 (87%)
Annual income below poverty line‡	6220	1144 (18%)	7075	1074 (15%)	765	690 (90%)	989	847 (86%)
Parental intermarriage	6207	253 (4%)	7076	249 (4%)	776	31 (4%)	1000	25 (3%)
**Clinical history**								
History of diabetes	7118	1258 (18%)	7114	728 (10%)	1005	287 (29%)	1006	109 (11%)
History of hypertension	7118	1710 (24%)	7114	898 (13%)	1004	460 (46%)	1006	206 (20%)
Waist-to-hip ratio	5955	0.98 (0.94, 1.02)	7112	0.97 (0.92, 1.01)	734	0.94 (0.90, 0.98)	1007	0.92 (0.87, 0.97)
**Lifestyle factors**								
Tobacco consumption	6278		7085		793		999	
Never	6278	847 (13%)	7085	2182 (31%)	793	324 (41%)	999	416 (42%)
Ex	6278	434 (7%)	7085	575 (8%)	793	30 (4%)	999	16 (2%)
Current	6278	4997 (80%)	7085	4328 (61%)	793	439 (55%)	999	567 (57%)
Amount of physical activity	7127		7116		1006		1008	
<600 MET/wk	7127	2890 (41%)	7116	1615 (23%)	1006	562 (56%)	1008	257 (25%)
>=600 MET/wk	7127	4237 (59%)	7116	5501 (77%)	1006	444 (44%)	1008	751 (75%)
**Blood lipids**								
Total cholesterol (mmol/l)	7115	4.96 (4.30, 5.70)	7108	4.68 (4.11, 5.35)	1003	5.36 (4.55, 6.14)	1007	4.97 (4.35, 5.65)
LDL-C (mmol/l)	7115	3.14 (2.55, 3.78)	7108	2.74 (2.21, 3.31)	1003	3.47 (2.77, 4.25)	1007	2.97 (2.46, 3.61)
HDL-C (mmol/l)	7115	0.79 (0.66, 0.93)	7108	0.81 (0.68, 0.96)	1003	0.91 (0.76, 1.08)	1007	0.95 (0.80, 1.14)

IQR, interquartile range; MI, myocardial infarction; MET, metabolic equivalents; HDL-C, high-density lipoprotein cholesterol; LDL-C, low-density lipoprotein cholesterol.

‡Less than 56,000 Taka according to World Health Organization defined poverty line ($1.90/day); 1 Taka = $0.012.

eTable 3. Participants characteristics by location and MI case-control status

	Urban				Rural			
	MI Cases		Controls		MI Cases		Controls	
Characteristics	N	Median (IQR) or n (%)	N	Median (IQR) or n (%)	N	Median (IQR) or n (%)	N	Median (IQR) or n (%)
**Socio-demographic factors**								
Age (yrs)	3305	50.0 (44.0, 58.0)	3625	50.0 (43.0, 56.0)	3690	55.0 (47.0, 60.0)	4427	52.0 (45.0, 60.0)
Male sex	3305	2994 (91%)	3625	3248 (90%)	3690	3225 (87%)	4427	3803 (86%)
Location	3305		3625		3690		4427	
Urban	3305	2213 (67%)	3625	2376 (66%)	3690	0 (0%)	4427	0 (0%)
Semi-urban	3305	1092 (33%)	3625	1249 (34%)	3690	0 (0%)	4427	0 (0%)
Rural	3305	0 (0%)	3625	0 (0%)	3690	3690 (100%)	4427	4427 (100%)
Education	3305		3625		3690		4427	
No formal education	3305	916 (28%)	3625	962 (27%)	3690	1718 (47%)	4427	1838 (42%)
Primary	3305	971 (29%)	3625	1031 (28%)	3690	1160 (31%)	4427	1365 (31%)
Secondary	3305	827 (25%)	3625	984 (27%)	3690	618 (17%)	4427	924 (21%)
Vocational/University	3305	591 (18%)	3625	648 (18%)	3690	194 (5%)	4427	300 (7%)
Occupation	3304		3625		3689		4426	
Business/professional	3304	1873 (57%)	3625	2006 (55%)	3689	1333 (36%)	4426	1531 (35%)
Industrial worker	3304	284 (9%)	3625	263 (7%)	3689	209 (6%)	4426	171 (4%)
Manual labour	3304	232 (7%)	3625	369 (10%)	3689	776 (21%)	4426	1297 (29%)
Unemployed/retired/student	3304	915 (28%)	3625	987 (27%)	3689	1371 (37%)	4426	1427 (32%)
Annual income below poverty line‡	3290	579 (18%)	3616	620 (17%)	3669	1244 (34%)	4407	1290 (29%)
Parental intermarriage	3288	129 (4%)	3614	153 (4%)	3674	154 (4%)	4425	120 (3%)
**Clinical history**								
History of diabetes	3305	714 (22%)	3623	435 (12%)	3690	578 (16%)	4426	397 (9%)
History of hypertension	3305	940 (28%)	3623	566 (16%)	3689	890 (24%)	4426	527 (12%)
Waist-to-hip ratio	3065	0.98 (0.94, 1.02)	3622	0.97 (0.92, 1.01)	3422	0.97 (0.92, 1.02)	4425	0.95 (0.90, 1.00)
**Lifestyle factors**								
Tobacco consumption	3302		3622		3690		4416	
Never	3302	628 (19%)	3622	1329 (37%)	3690	517 (14%)	4416	1259 (29%)
Ex	3302	220 (7%)	3622	303 (8%)	3690	241 (7%)	4416	283 (6%)
Current	3302	2454 (74%)	3622	1990 (55%)	3690	2932 (79%)	4416	2874 (65%)
Amount of physical activity	3305		3625		3690		4427	
<600 MET/wk	3305	1239 (37%)	3625	962 (27%)	3690	1101 (30%)	4427	868 (20%)
>=600 MET/wk	3305	2066 (63%)	3625	2663 (73%)	3690	2589 (70%)	4427	3559 (80%)
**Blood lipids**								
Total cholesterol (mmol/l)	3299	5.02 (4.37, 5.76)	3619	4.68 (4.11, 5.37)	3681	4.98 (4.28, 5.75)	4424	4.74 (4.16, 5.41)
LDL-C (mmol/l)	3299	3.17 (2.58, 3.81)	3619	2.76 (2.23, 3.34)	3681	3.16 (2.56, 3.84)	4424	2.78 (2.24, 3.36)
HDL-C (mmol/l)	3299	0.78 (0.66, 0.92)	3619	0.82 (0.68, 0.97)	3681	0.82 (0.69, 0.97)	4424	0.83 (0.70, 0.99)

IQR, interquartile range; MI, myocardial infarction; MET, metabolic equivalents; HDL-C, high-density lipoprotein cholesterol; LDL-C, low-density lipoprotein cholesterol.

‡Less than 56,000 Taka according to World Health Organization defined poverty line ($1.90/day); 1 Taka = $0.012.

eFigure 1. Associations of additional factors with MI

	N of Cases	OR (95% CI)	P value
Annual income below poverty line	6965	1.25 (1.13, 1.38)	<0.0001
Formal education <5 years	7001	1.29 (1.19, 1.39)	<0.0001
Occupation (*vs* Business/professional)	7012		
Industrial worker		1.40 (1.21, 1.62)	<0.0001
Manual labour		0.73 (0.66, 0.80)	<0.0001
Unemployed/retired/student		1.05 (0.96, 1.15)	0.28
Lifelong urban residence	6976	1.38 (1.22, 1.56)	<0.0001
Parental intermarriage	6964	1.26 (1.05, 1.51)	0.013
Often or always stressed at home	6982	1.15 (1.07, 1.23)	0.0002
Often or always stressed at work	5648	1.37 (1.25, 1.50)	<0.0001
Daytime nap ≥ 1hr/day	6802	1.17 (1.08, 1.26)	<0.0001

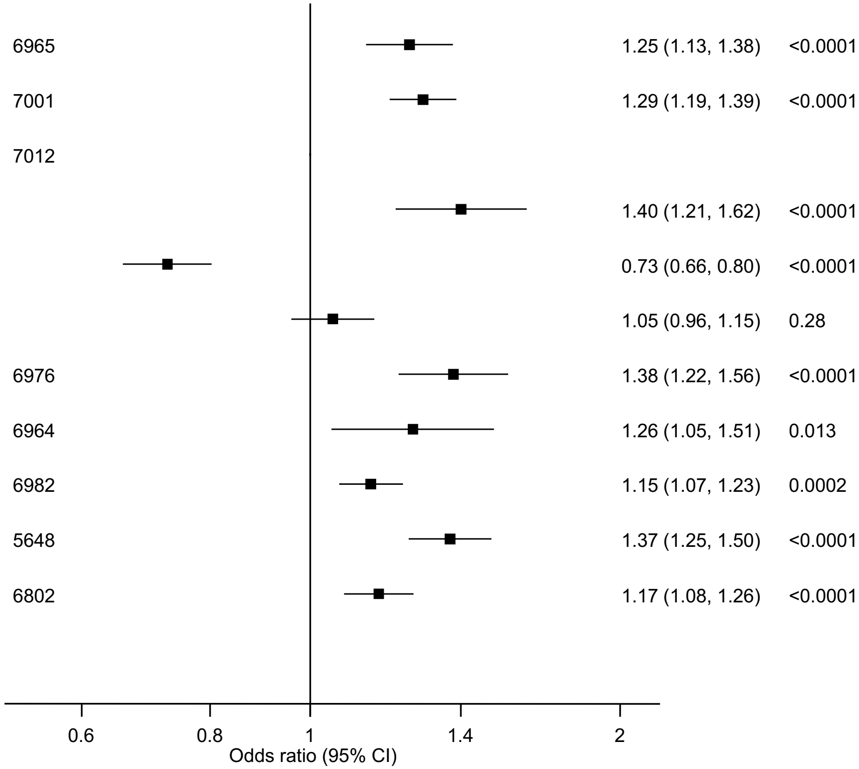

Analysis adjusted for age, sex, tobacco use, LDL-cholesterol, history of diabetes, history of hypertension, and family history of MI.

eFigure 2. Physical activity and risk of MI

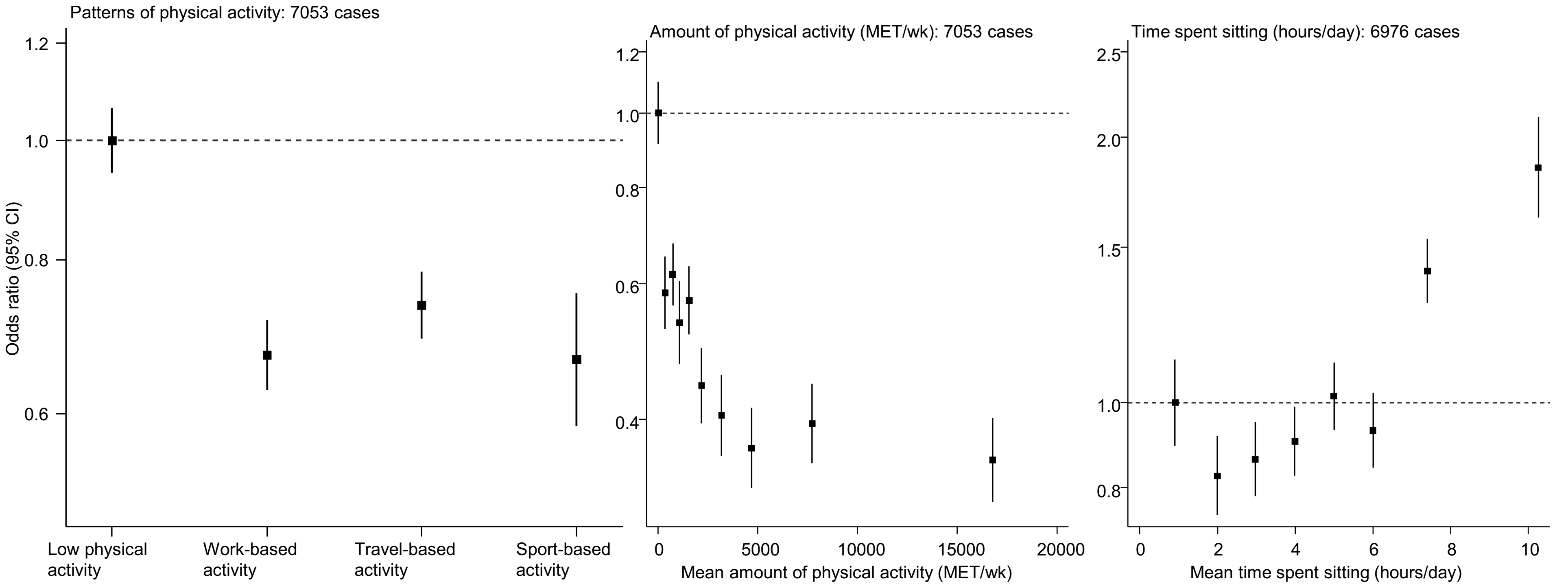

Analysis adjusted for age, sex, tobacco use, LDL-cholesterol, history of diabetes, history of hypertension, and family history of MI.

MET, metabolic equivalents

eFigure 3. Association of food groups with MI.

	N of Cases	OR (95% CI)	P value
Total energy intake	6982	0.52 (0.47, 0.59)	<0.0001
		0.52 (0.47, 0.59)	<0.0001
Total oil	6915	1.15 (1.03, 1.29)	0.014
		1.25 (1.12, 1.40)	0.0001
Rice	6982	0.79 (0.71, 0.88)	<0.0001
		1.02 (0.90, 1.15)	0.74
Bread	6981	0.83 (0.74, 0.94)	0.002
		0.96 (0.85, 1.08)	0.50
Red meat	6981	0.84 (0.75, 0.93)	0.0013
		1.00 (0.89, 1.12)	0.99
Poultry	6980	0.76 (0.68, 0.84)	<0.0001
		0.90 (0.81, 1.01)	0.069
Dairy	6977	0.83 (0.74, 0.92)	0.0004
		1.01 (0.91, 1.13)	0.83
Fish	6979	0.97 (0.87, 1.08)	0.57
		1.55 (1.37, 1.77)	<0.0001
Pulses	6977	0.81 (0.72, 0.90)	0.0001
		1.04 (0.92, 1.17)	0.54
Vegetables	6979	0.51 (0.46, 0.57)	<0.0001
		0.62 (0.55, 0.70)	<0.0001
Fruit	6978	0.44 (0.40, 0.49)	<0.0001
		0.52 (0.46, 0.59)	<0.0001

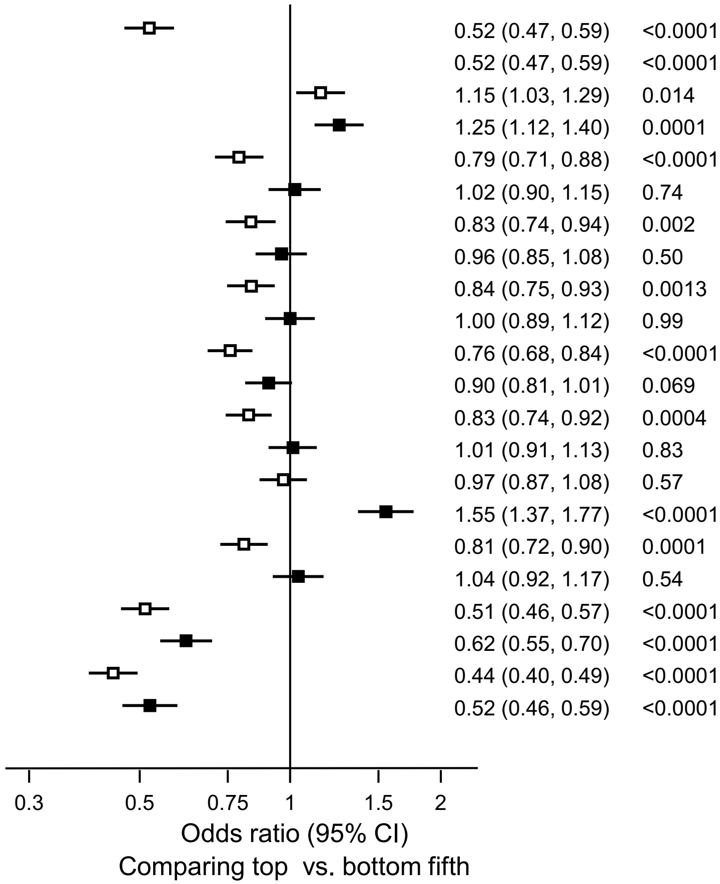

Adjusted for age, sex, smoking, history of diabetes, history of hypertension, family history of MI, and LDL-C

Further adjusted for total energy intake

eFigure 4. Sensitivity analysis with multiple imputation of missing data: Prevalences, absolute and relative risk estimates for various conventional risk factors with MI.

	N of Controls	N of Cases	Prevalence (%) or mean(SD) Controls	Cases	OR (95% CI)	PAF (95% CI)
History of diabetes	8124	8133	10.3	19.0	1.98 (1.79, 2.19)	9.4 (8.4, 10.4)
History of hypertension	8124	8133	13.6	26.7	2.29 (2.09, 2.50)	15.0 (14.0, 16.1)
Family history of MI	8124	8133	7.2	13.7	1.88 (1.68, 2.10)	6.4 (5.6, 7.3)
Smoking status	8124	8133				
Current vs never	4922	6223	60.6	76.5	2.76 (2.54, 3.01)	48.9 (46.5, 51.2)
Former vs never	597	547	7.3	6.7	1.57 (1.34, 1.83)	2.4 (1.7, 3.0)
Waist:Hip ratio						
SD higher	8124	8133	0.96 (0.07)	0.97 (0.07)	1.13 (1.08, 1.18)	
Top vs bottom third	2443	3043	30.1	37.4	1.35 (1.24, 1.47)	9.6 (7.2, 12.0)
Middle vs bottom third	2598	2793	32.0	34.3	1.29 (1.19, 1.40)	7.7 (5.4, 9.9)
Total cholesterol (mmol/l)						
SD higher	8124	8133	4.80 (1.03)	5.11 (1.15)	1.32 (1.28, 1.37)	
Top vs bottom third	2706	3669	33.3	45.1	1.76 (1.63, 1.91)	19.5 (17.4, 21.5)
Middle vs bottom third	2687	2390	33.1	29.4	1.16 (1.07, 1.26)	4.0 (1.9, 6.1)
LDL-C (mmol/l)						
SD higher	8124	8133	2.81 (0.88)	3.23 (1.04)	1.53 (1.48, 1.58)	
Top vs bottom third	2687	4148	33.1	51.0	2.58 (2.38, 2.80)	31.2 (29.6, 32.8)
Middle vs bottom third	2703	2318	33.3	28.5	1.43 (1.31, 1.56)	8.6 (6.8, 10.3)
HDL-C (mmol/l)						
SD lower	8124	8133	0.85 (0.23)	0.82 (0.22)	1.23 (1.19, 1.28)	
Bottom vs top third	2851	3137	35.1	38.6	1.49 (1.37, 1.62)	12.6 (10.4, 14.8)
Middle vs top third	2655	2668	32.7	32.8	1.17 (1.07, 1.27)	4.7 (2.3, 7.0)

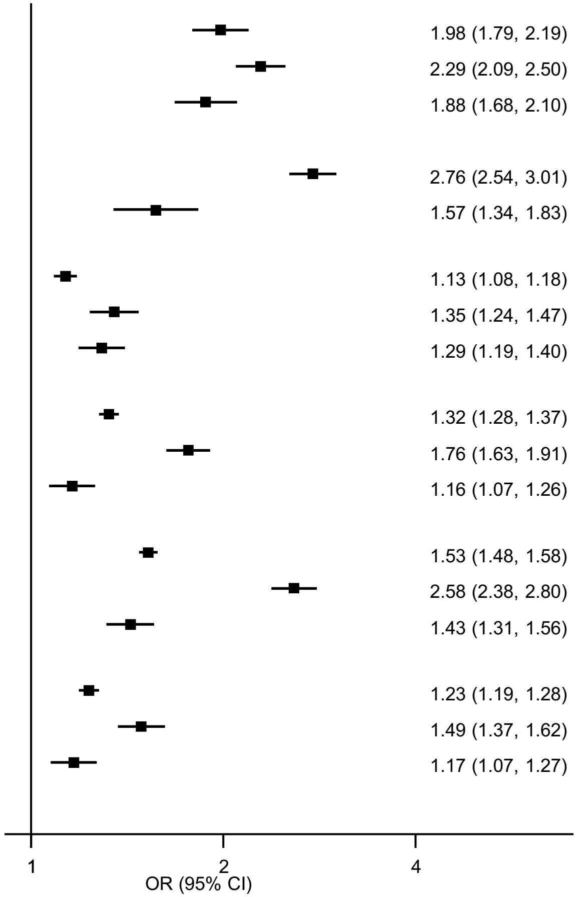

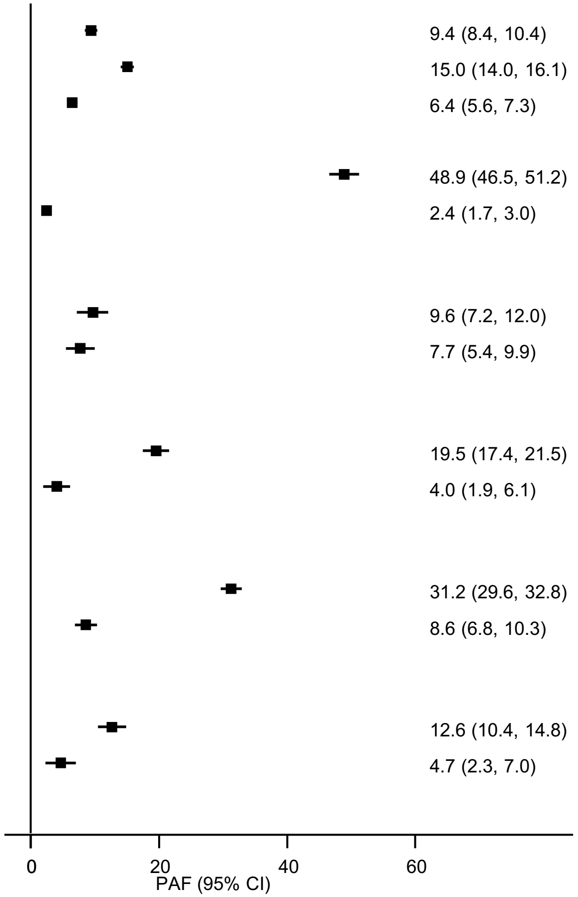

Analysis adjusted for age, sex, tobacco use, LDL-cholesterol, history of diabetes, history of hypertension, and family history of MI.

SD, standard deviation; HDL-C, high-density lipoprotein cholesterol; LDL-C, low-density lipoprotein cholesterol.

eFigure 5. Sensitivity analysis with multiple imputation of missing data: Associations of various conventional risk factors with MI, stratified by age, sex and location.

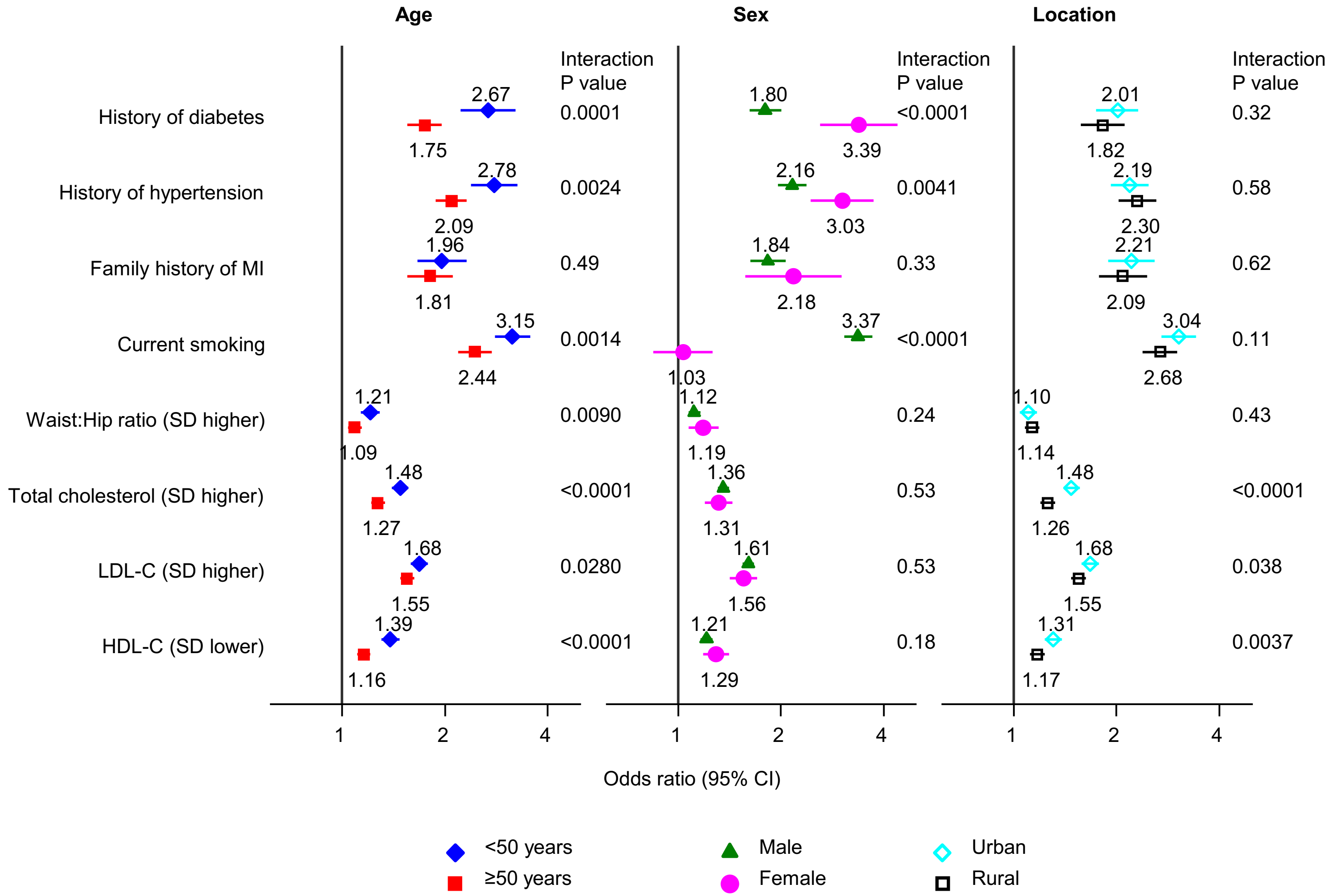

Analysis adjusted for age, sex, tobacco use, LDL-cholesterol, history of diabetes, history of hypertension, and family history of MI.

eFigure 6. Sensitivity analysis with multiple imputation of missing data: Associations of additional factors with MI.

	N of Cases	OR (95% CI)	P value
Annual income below poverty line	8133	1.25 (1.13, 1.38)	<0.0001
Formal education <5 years	8133	1.28 (1.19, 1.38)	<0.0001
Occupation (vs Business/professional)	8133		
Industrial worker		1.39 (1.20, 1.61)	<0.0001
Manual labour		0.72 (0.65, 0.80)	<0.0001
Unemployed/retired/student		1.05 (0.96, 1.16)	0.26
Lifelong urban residence	8133	1.38 (1.22, 1.56)	<0.0001
Parental intermarriage	8133	1.25 (1.04, 1.49)	0.017
Often or always stressed at home	8133	1.14 (1.06, 1.23)	0.0002
Often or always stressed at work	8133	1.37 (1.25, 1.50)	<0.0001
Daytime nap >= 1hr/day	8133	1.16 (1.08, 1.26)	0.0001

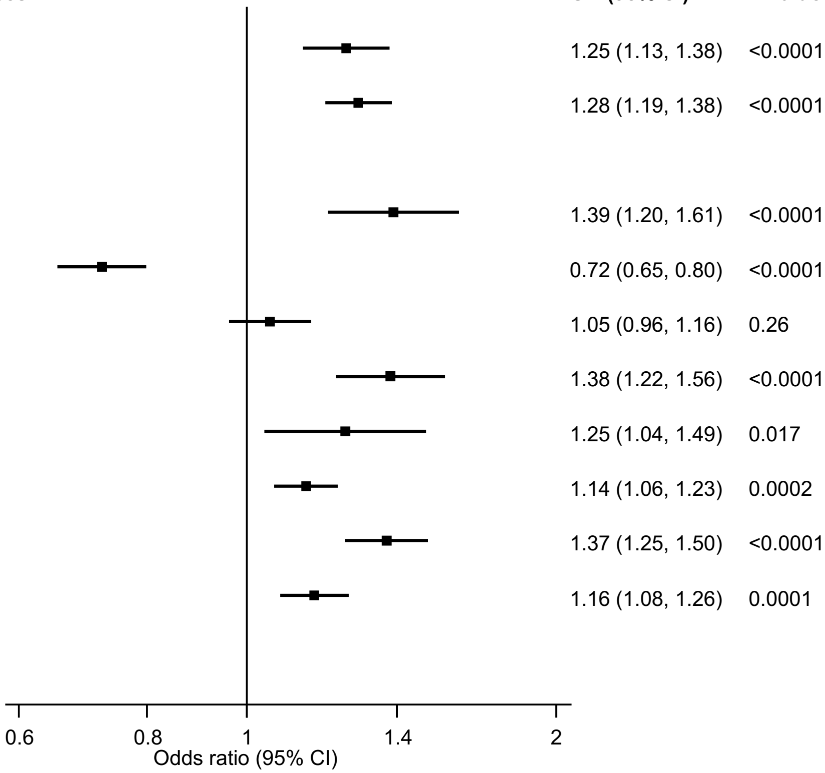

Analysis adjusted for age, sex, tobacco use, LDL-cholesterol, history of diabetes, history of hypertension, and family history of MI.

eFigure 7. Sensitivity analysis with multiple imputation of missing data: Associations of food groups with MI.

	N of Cases	OR (95% CI)	P value
Total energy intake	8133	0.52 (0.47, 0.58)	<0.0001
		0.52 (0.47, 0.58)	<0.0001
Total oil	8133	1.15 (1.03, 1.29)	0.014
		1.25 (1.12, 1.40)	0.0001
Rice	8133	0.79 (0.70, 0.88)	<0.0001
		1.02 (0.90, 1.15)	0.76
Bread	8133	0.84 (0.75, 0.94)	0.0027
		0.97 (0.86, 1.09)	0.56
Red meat	8133	0.84 (0.75, 0.93)	0.0014
		1.00 (0.89, 1.12)	0.99
Poultry	8133	0.76 (0.68, 0.84)	<0.0001
		0.91 (0.81, 1.01)	0.072
Dairy	8133	0.82 (0.74, 0.92)	0.0003
		1.01 (0.90, 1.13)	0.85
Fish	8133	0.97 (0.87, 1.08)	0.58
		1.55 (1.37, 1.77)	<0.0001
Pulses	8133	0.81 (0.72, 0.90)	0.0001
		1.04 (0.92, 1.17)	0.54
Vegetables	8133	0.51 (0.46, 0.57)	<0.0001
		0.62 (0.55, 0.70)	<0.0001
Fruit	8133	0.44 (0.40, 0.50)	<0.0001
		0.53 (0.46, 0.60)	<0.0001

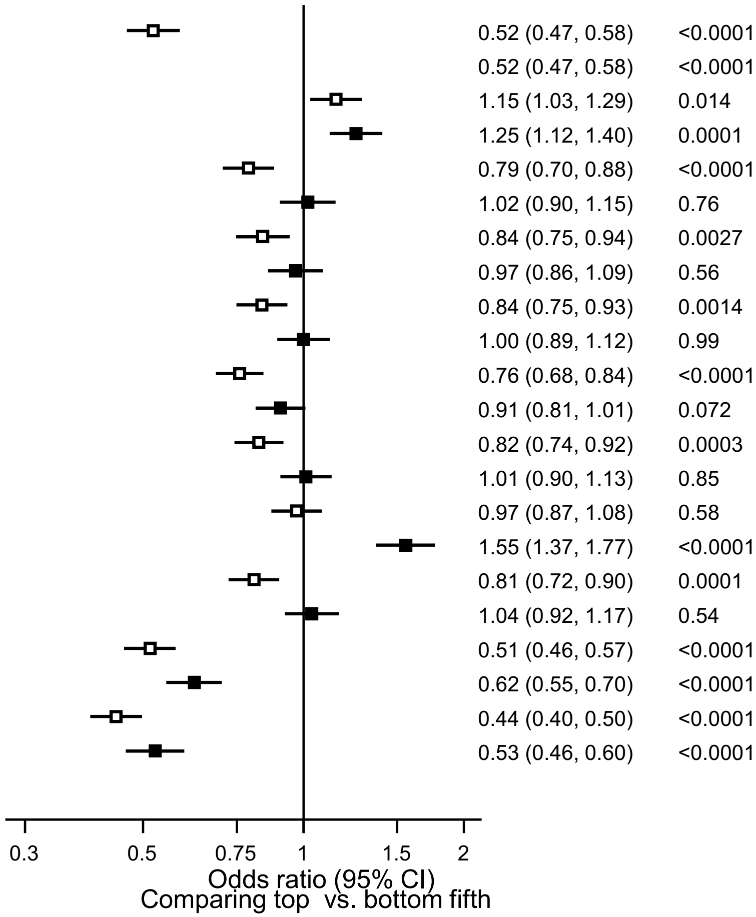

□ Adjusted for age, sex, smoking, history of diabetes, history of hypertension, family history of MI, and LDL-C

■ Further adjusted for total energy intake
